# Microcephaly measurement in adults and its association with clinical variables

**DOI:** 10.11606/s1518-8787.2022056004175

**Published:** 2022-05-18

**Authors:** Nicole Rezende da Costa, Livia Mancine, Rogerio Salvini, Juliana de Melo Teixeira, Roberta Diehl Rodriguez, Renata Elaine Paraizo Leite, Camila Nascimento, Carlos Augusto Pasqualucci, Ricardo Nitrini, Wilson Jacob-Filho, Beny Lafer, Lea Tenenholz Grinberg, Claudia Kimie Suemoto, Paula Villela Nunes

**Affiliations:** I Universidade de São Paulo Faculdade de Medicina São Paulo SP Brasil Universidade de São Paulo. Faculdade de Medicina. São Paulo, SP, Brasil; II Universidade Federal de Goiás Instituto de Informática Goiânia GO Brasil Universidade Federal de Goiás. Instituto de Informática. Goiânia, GO, Brasil; III Instituto Federal Goiano Departamento de Ensino Iporá GO Brasil Instituto Federal Goiano. Departamento de Ensino. Iporá, GO, Brasil; IV University of California Memory and Aging Center San Francisco California United States University of California. Memory and Aging Center. San Francisco, California, United States

**Keywords:** Adult, Microcephaly, classification, Cephalometry, Dementia, Data Mining

## Abstract

**OBJECTIVE:**

To establish a microcephaly cut-off size in adults using head circumference as an indirect measure of brain size, as well as to explore factors associated with microcephaly via data mining.

**METHODS:**

In autopsy studies, head circumference was measured with an inelastic tape placed around the skull. Total brain volume was also directly measured. A linear regression was used to determine the association of head circumference with brain volume and clinical variables. Microcephaly was defined as head circumference that were two standard deviations below the mean of significant clinical variables. We further applied an association rule mining to find rules associating microcephaly with several sociodemographic and clinical variables.

**RESULTS:**

In our sample of 2,508 adults, the mean head circumference was 55.3 ± 2.7cm. Head circumference was related to height, cerebral volume, and sex (p < 0.001 for all). Microcephaly was present in 4.7% of the sample (n = 119). Out of 34,355 association rules, we found significant relationships between microcephaly and a clinical dementia rating (CDR) > 0.5 with an informant questionnaire on cognitive decline in the elderly (IQCODE) ≥ 3.4 (confidence: 100% and lift: 5.6), between microcephaly and a CDR > 0.5 with age over 70 years (confidence: 42% and lift: 2.4), and microcephaly and males (confidence: 68.1% and lift: 1.3).

**CONCLUSION:**

Head circumference was related to cerebral volume. Due to its low cost and easy use, head circumference can be used as a screening test for microcephaly, adjusting it for gender and height. Microcephaly was associated with dementia at old age.

## INTRODUCTION

Head circumference (HC) is an anthropometric parameter highly correlated with brain size^1 , 2^. A usual standard for microcephaly is an HC more than two standard deviations below the mean^3^. Microcephaly can be present at birth - primary microcephaly - or develop postnatally - secondary microcephaly^6^. Some causes of congenital microcephaly are genetic mutations, whereas other modifiable causes include prenatal infections (e.g., exposure to the Zika virus), maternal alcohol and substance abuse, and inadequate nutrition during pregnancy^7^. Secondary microcephaly can occur due to deceleration of brain growth in face of infection, trauma, intoxication, metabolic disease, and Rett syndrome, among other examples^4^. Moreover, microcephaly may lead to various developmental abnormalities and decreased cognitive reserve, with long-term consequences such as the increased risk for forms of dementia in vulnerable individuals^8^.

Microcephaly parameters are well established for children aged 0 to 18 months^11^, and the World Health Organization makes available charts for the HC growth in children from birth to the age of five years, plotted as standard deviations from the mean^12^. Nevertheless, even though HC is an accessible and inexpensive measure, we lack defined parameters for microcephaly in adults, and little is known about the clinical implications of microcephaly in this population.

This study aimed to establish a microcephaly cut-off size in adults using HC as an indirect measure of brain volume, as well as to investigate factors related to microcephaly via data mining to elicit several possible associations.

## METHODS

### Data Source

This cross-sectional study was conducted on subjects who underwent autopsy at the São Paulo Autopsy Service (SVOC-USP) between 2004 and 2019. In Brazil, autopsies are mandatory for all individuals whose cause of death was unidentified after death. The SVOC-USP is a community-based general autopsy service.

### Study Population

Data were derived from the collection of the Biobank for Aging Studies at the Universidade de São Paulo (BAS-USP). Our study protocol was reviewed and approved by the ethics committee of the *Faculdade de Medicina* at the *Universidade de São Paulo* (approval number 458.272), following the World Medical Association Declaration of Helsinki. Subjects were included in our study after its procedures had been explained to family members and they had signed an informed consent form, thus agreeing to participate in our research.

The methodological procedures of the BAS-USP have been described elsewhere^13^. Subjects who were aged 50 years or older and who had died from natural (non-traumatic) causes were included. Cases without reliable informants, with a medical history of advanced chronic diseases or a prolonged agonal state were excluded. Subjects with significant cerebral lesions, including stroke and cerebral tumors, were excluded from the BAS-USP cohort because an immediate brain examination is required to confirm the cause of death. Nurses with expertise in gerontology invited knowledgeable informants to participate in our study. Knowledgeable informants were close family members or caregivers who had at least weekly contact with the deceased in the last six months before their death and could recount and provide details on subjects’ health information.

### Clinical Post-Mortem Evaluation

Clinical evaluation consisted of assessing subjects’ clinical and functional status three months before death. A validated semi-structured clinical interview^16^ assessed demographic variables (age, sex, and educational attainment), conditions related to death, past medical history, and cognitive status. For the association rule mining (ARM), age was also categorized according to the median of the sample as < 70 and ≥ 70 years old, and education was stratified into illiterate, 1–4 years of study, and five years or more. Cognitive status in the three months before death was assessed by the informant questionnaire on cognitive decline in the elderly (IQCODE)^17^, and informants aided in the clinical dementia rating (CDR)^18^, validated for post-mortem use^16^. The IQCODE assesses the cognitive decline in the elderly in the past ten years, and the IQCODE cut-off was ≥ 3.4^19^. The CDR was used to identify the presence and stages of dementia, and a CDR > 0.5 was considered indicative of cognitive impairment^18^.

Clinical medical history was assessed in detail during the interviews with informants, including history of hypertension, diabetes mellitus, coronary artery disease, congestive heart failure, dyslipidemia, cardiac arrhythmia, stroke, alcohol abuse/alcoholism, and tobacco use.

During the clinical evaluation, the interviewer continuously checked for data consistency and exclusion criteria to detect any conditions that might lead to the exclusion of the case at hand.

### Morphometric Measurements

Head circumference was obtained before opening the skull. An inelastic tape was placed around the skull to obtain the largest perimeter when across the glabella and opstocranium^2 , 20^. Brain volume (in mL) was obtained by estimating the volume of water displaced by the submerged brain, according to Archimedes’ principle, a standard procedure for accurately measuring the volume of body regions^21^.

In this study, the microcephaly cut-off was defined in two steps. First, we analyzed which variables were associated with different HC measures. The variables tested were sex, height, age, and educational attainment. Second, microcephaly was set at two standard deviations below the mean HC for each group of clinical variables found to have a correlation with HC.

### Statistical Analysis

Spearman correlation test was used to determine the association of HC with brain volume, height, age, and education. Moreover, differences in HC between sexes were tested with independent sample t-Tests. The significant associations or differences found were included in a multivariate linear regression analysis. The entire sample was divided into quartile measures of height to obtain the HC adjusted for height, with 10cm divisions; a sample division followed this step according to sex. The level of significance of the two-tailed tests was set at 0.05. The software Stata 12.0 (College Station, TX: StataCorp LP) was used to perform the statistical analyses.

### Association Rule Mining

Association rule mining is a suitable method for discovering patterns or extracting co-occurrences of events from databases. It is a rule-based machine learning method for discovering multiple concomitant relations between variables in large databases. We can derive association rules from the frequency of variable sets, called itemsets, in an ordinal data set. An item is any variable characterizing a particular individual. A frequent itemset is any set of items with a frequency greater than or equal to a user’s predefined minimum threshold^22^.

An association rule has the form (X ⇒ Y) with the logical meaning “IF X, THEN Y”; in which X and Y are sets of non-overlapping items, i.e., X implies the occurrence of Y. X and Y are called the antecedent and consequent of the rule, respectively.

Association rule mining is probabilistic, and its primary measure assessments are values for support, confidence, and lift. Support is defined by the joint probability of X and Y in the data set, i.e., the percentage of records containing X and Y (Equation 1).


$$\text{ Equation 1: Support }(X\Rightarrow Y)=\frac{\text{ Occurrences of both }X\wedge Y}{\text{ Total number of records }}$$

Confidence is defined by the conditional probability of Y occurring given X in the data set, i.e., the percentage of times both X and Y occur (Equation 2).


$$\text{ Equation 2: Confidence }(X\Rightarrow Y)=\frac{\text{ Occurrences ofboth }X\wedge Y}{\text{ Occurences of }X}$$

The lift measures the dependency relationship between X and Y (Equation 3) by assessing how many times more often X and Y occur together than expected if they were statistically independent. A lift value of one indicates X and Y are independent. A lift value greater than one means that X and Y are positively correlated, and a lift value lower than one, that X and Y are negatively correlated.


$$\text{ Equation 3: Lift }(X\Rightarrow Y)=\frac{\text{ Occurrences of both }X\wedge Y}{\text{ Occurrences of }X\times \text{ Occurrences of }Y}$$

Association rule mining aims to discover frequent and reliable association rules, i.e., rules with user-specified minimum thresholds of support and confidence. Additionally, it can also specify the maximum size of a rule, defined as the number of items comprising it. For example, a rule of size three means that it consists of two items in the antecedent and one item in the consequent, whereas a rule of size two means that it consists of one item in the antecedent and one item in the consequent.

The most used ARM is the Apriori algorithm, introduced by Agrawal et al.^22^ in 1993. It consists of two steps. In the first step, frequent patterns (itemsets with support greater than the predefined minimal support) are generated. In the second step, frequent pattern confidences are estimated, and those with confidence greater than the set minimal confidence are selected as the final rules. In this study, a 1% minimum support and a 30% minimum confidence were established. These thresholds were chosen due to the low frequency of microcephaly in the data set, thus requiring lower values to obtain microcephaly associations. Moreover, we set the maximum size of a rule as three; this meant that the generated rules were either size two or three. This rule size was chosen to help us interpret the associations since, with more extensive rules, it would be more challenging to analyse how microcephaly is associated with other variables. The package *arules* of the R language was used to perform the Apriori algorithms.

### Ethics

Study approval statement: the data was derived from the collection of the Biobank for Aging Studies at the Universidade de São Paulo (BAS-USP). This study protocol was reviewed and approved by the ethics committee of the Escola de Medicina at the Universidade de São Paulo (approval number 458.272), following the World Medical Association Declaration of Helsinki.

Consent statement: a knowledgeable informant was a close family member or caregiver who had at least weekly contact with the deceased in the last six months before their death and could recount and provide details on the deceased’s health information.

## RESULTS

From 2004 to 2019, we obtained data from 2,508 individuals. Mean age was 70.8 (± 12.7) years, and average education, 4.7 (± 3.8) years. Mean brain volume was 1,169 (± 163) mL; mean HC, 55.3 (± 2.7) cm; and mean height, 1.68 (±10.3) m. We found smaller brain volumes in individuals with a CDR > 0.5 than in individuals with a CDR ≤ 0.5 (1,090 ± 160; 1,179 ± 161 mL, respectively, p < 0.001). Table 1 shows the categorized sociodemographic and clinical variables for the ARM.

**Table 1:** Sociodemographic and clinical variables of the sample (n = 2,508).

Covariables	n (%)
Age ≥ 70 years	1,349 (53.8)
Female	1,195 (47.7)
Education	394 (15.8)
Illiterate	
1–4 years	1,368 (54.8)
≥ 5 years	734 (29.4)
Hypertension	1,482 (64.7)
Diabetes mellitus	696 (27.8)
Coronary artery disease	511 (20.4)
Congestive heart failure	422 (16.8)
Dyslipidemia	244 (9.7)
Cardiac arrhythmia	174 (6.9)
Stroke	298 (11.9)
Alcohol abuse/alcoholism	375 (15.0)
Tobacco use	790 (31.5)
CDR > 0.5	436 (17.5)
IQCODE ≥ 3.4	446 (17.9)

CDR > 0.5: clinical dementia rating indicative of cognitive impairment; IQCODE ≥ 3.4: cut-off for the informant questionnaire on cognitive decline in the elderly in the past 10 years.

Head circumference was correlated to brain volume (rho = 0.466, p < 0.001), height (rho = 0.394, p < 0.001), age (rho = -0.25, p < 0.001), and education (rho = 0.169, p < 0.001), and differed between sexes (men = 56.3 ±2 .6 cm, women = 54.2 ± 2.4 cm, p < 0.001).

In the multivariate analysis with brain volume, sex, height, age, and education as covariables, HC was related to brain volume, sex, height, but not age or education, as Table 2 shows. If we excluded cases with a CDR > 0.5 (as HC remains constant, but brain volume can atrophy), the significance of brain volume, sex, and height would have remained p < 0.001. Table 3 shows the number of participants with microcephaly according to height and sex. If we consider microcephaly to be an HC two standard deviations below the mean, according to height and sex, it was present in 4.7% of the sample (n = 119).

**Table 2:** Association of head circumference with sociodemographic and clinical variables (n = 2,508).

Covariables	β (95%CI)	p
Brain volume (mL)	0.005 (0.005 to 0.006)	**< 0.001**
Age (years)	-0.009 (-0.020 to 0.002)	0.10
Sex (female)	0.849 (0.573 to 1.124)	**< 0.001**
Education (years)	0.012 (-0.019 to 0.044)	0.44
Height (cm)^a^	0.043 (0.029 to 0.058)	**< 0.001**

^a^ Multivariate linear regression analysis.

**Table 3:** Head circumference and microcephaly cut-off values according to sex and height (n = 2,508).

Distribution of height (m)	Female			Male		
	n	HC (cm), mean ± SD	Microcephaly cut-off (cm)	n	HC (cm)	Microcephaly cut-off (cm)
Up to 1.59	282	53.5 ± 2.4	< 48.7	46	55.2 ± 2.3	< 50.6
1.60–1.69	301	54.3 ± 2.2	< 49.9	198	55.4 ± 2.4	< 51.0
1.70–1.79	327	54.7 ± 2.2	< 50.3	427	56.2 ± 2.4	< 51.8
1.8 or more	45	55.6 ± 2.6	< 50.4	374	56.7 ± 2.5	< 51.7

HC: head circumference.

### Association Rules with Microcephaly

All variables assessed, via ARM, for their relationship to microcephaly were ordinal. Those variables included age, sex, education, hypertension, diabetes mellitus, coronary artery disease, congestive heart failure, dyslipidemia, cardiac arrhythmia, stroke, alcohol abuse/alcoholism, tobacco use, a CDR > 0.5, and an IQCODE ≥ 3.4. The ARM produced 34,355 candidate rules that satisfied the required thresholds in the discovery data set. Then, we selected only the rules which included microcephaly to analyze possible associations with the other variables, resulting in 258 association rules. Finally, we evaluated the rules with the highest degree of association between microcephaly and the other variables by their lift values.

### Analysis of the Rules of Size Three

Initially, we analyzed the rules of size three, i.e., rules with associations between three variables, one of them being microcephaly. Three rules had lift values of more than two, expressing the most significant associations: IF a CDR > 0.5 AND microcephaly, THEN an IQCODE ≥ 3.4 (confidence: 100%, lift: 5.6); IF an IQCODE ≥ 3.4 AND microcephaly, THEN a CDR > 0.5 (confidence: 100%, lift: 5.6); and IF age over 70 years AND microcephaly, THEN CDR > 0.5 (confidence: 42%, lift: 2.4).

After this initial analysis, we sought rules which excluded microcephaly to investigate how it influenced these associations. Table 4 shows a comparison between the association rules with and without microcephaly. Confidence and lift values were higher if microcephaly were present in the association.

**Table 4:** Comparison between rules that show associations among CDR > 0.5, IQCODE ≥ 3.4, and age over 70 years with and without the presence of microcephaly.

Rules with microcephaly	Confidence	Lift	Rules without microcephaly	Confidence	Lift
IF CDR > 0.5 AND microcephaly, THEN IQCODE ≥ 3.4	100%	5.6	IF CDR > 0.5 THEN IQCODE ≥ 3.4	95.4%	5.4
IF IQCODE ≥ 3.4 AND microcephaly, THEN CDR > 0.5	100%	5.6	IF IQCODE≥3.4 THEN CDR>0.5	93.3%	5.4
IF age over 70 years AND microcephaly, THEN CDR > 0.5	42.0%	2.4	IF age over 70 years THEN CDR > 0.5	28.4%	1.6

CDR > 0.5: clinical dementia rating indicative of cognitive impairment; IQCODE ≥ 3.4: cut-off for the informant questionnaire on cognitive decline in the elderly in the past 10 years.

### Analysis of the Rules of Size Two

We also analysed rules of size two, finding significant associations between lift values greater than one and males: IF microcephaly, THEN male (confidence: 68.1%, lift: 1.3). Then, we examined the association between microcephaly and females: IF microcephaly, THEN female (confidence: 31.9%, lift: 0.7), in which lift values below one indicate a negative association between microcephaly and females.

Finally, besides the associations related to microcephaly (the focus of this study), other rules produced associations, such as IF diabetes, THEN hypertension (confidence: 81.8%, lift: 1.4); IF stroke, THEN diabetes (confidence: 39.1%, lift: 1.4); IF stroke, THEN hypertension (confidence: 80.2%, lift: 1.4); IF an IQCODE ≥ 3.4, THEN female (confidence: 63.7%, lift: 1.3); and IF a CDR > 0.5, THEN female (confidence: 63.5%, lift: 1.3).

## DISCUSSION

Our study is one of the few analyzing the relationship of microcephaly with clinical and sociodemographic variables and the only one that used ARM, a data mining approach. We found that microcephaly was associated with dementia or cognitive impairment, especially in individuals older than 70 years.

Head circumference correlated to adult brain volume without dementia, and it is a non-invasive, fast, and inexpensive method to indirectly measure child or adult brain volume. As HC remains constant across the life span, these results suggest that microcephaly might be a risk factor for dementia at old age, as structural changes in the brain may impact cognition across the older age span^23^.

The results of the association of microcephaly with a CDR > 0.5 and an IQCODE ≥ 3.4 agree with previous studies. A longitudinal study evaluating 1,569 individuals, aged 60 and over, from a Korean community showed that the clinical expression of dementia related to brain volume. People with larger brains were more likely to remain nondemented^24^. Another frequently cited longitudinal study, in which 294 catholic sisters were assessed annually for dementia, found that high educational attainment and larger head size, either by themselves or in combination, may reduce the risk of the expression of dementia in later life^25^. A population study based on the Well-being of the Singapore Elderly survey assessed associations between dementia, HC, and leg length among the older adult population and found that HC is independently associated with dementia among that population, suggesting that the risk factors for dementia exert their influence since early life^9^. With more neurons and synapses, maximum brain volume may be an important variable associated with brain reserve^20 , 24 , 26^.

Machine learning algorithms can complement classical statistics^27^, helping researchers to create new hypotheses^28^. We used ARM in our study for two main reasons. First, ARM enabled us to observe all associations among the clinic and sociodemographic variables available in our database. Second, we could verify, by the ARM metrics, how strong the associations were when we compared the rules in the presence or absence of microcephaly.

The significant association we found between microcephaly and males agrees with the literature, as mental retardation is more frequent in boys than girls, a finding attributed to mutations in X-linked genes^28 , 29^. Besides the associations related to microcephaly (the focus of this study), ARM also produced associations which are well established in the literature, such as the ones between diabetes and hypertension, stroke and diabetes, stroke and hypertension, cognitive decline and females, and dementia and females^30^, reinforcing the use of this method in showing reliable associations.

In our study, we found that HC correlated with brain volume, sex, and height. Individuals with dementia showed a smaller brain volume, an expected atrophy due to their condition. Measuring HC has advantages since it is a non-invasive, fast, and inexpensive method to indirectly measure child or adult brain volume. To indirectly determine microcephaly via HC, we must, ideally, consider sex and height. In our sample, men with an HC < 51cm and women with an HC < 49cm are indicative of microcephaly; if height > 1.7m, one should add 1cm to the HC. We also find significant associations of HC with brain volume in the few studies conducted in adults^1 , 2 , 31^. The relationship of HC with age and sex is well established in children aged 0 to 18 months^11^. In this study, we considered the corrections for sex and height, but not age, appropriate to determine microcephaly in adults, despite the century-long growth trend many countries show^34^ — attributed to improved environmental conditions^34^. As a result, we now have progressively larger adults than in previous decades. Taller people often have larger brains and heads^35 , 36^. In this study, however, after the logistic regression, height and sex, but not age, related to HC and brain size.

Strengths of our study include its large sample size, community basis, and an ethnically and educationally diverse population. However, our study has some limitations: its cross-sectional nature fails to allow for causal relationships. Moreover, the use of informant-reported data is a concern, as informants can be unaware of some of the treatments and disorders the deceased may have had. However, we used a validated semi-structured clinical interview^16^ which several other publications accept^13^. Our study assessed a community sample in Brazil. Samples from multiracial countries, such as ours, can add valuable data to the literature, but the validity of our findings to other populations needs further testing. Furthermore, to the best of our knowledge, this is the first study that applied ARM to detect rules associated with microcephaly in adults. This strategy has the advantage of setting high-accuracy standards and the analysis of multiple variables at the same time.

## CONCLUSION

This population-based cross-sectional study suggests that HC not only relates to cerebral volume but could also function as an accessible and inexpensive screening test for microcephaly, in conjunction with individuals’ height and sex. Moreover, we found an association between microcephaly and clinical variables often present in cognitive decline at older age which might be a risk factor for dementia.
